# Supplementary material for: Chemical Characterization and Metagenomic Identification of Endophytic Microbiome from South African Sunflower (Helianthus annus) Seeds
Source: Microorganisms. 2023 Apr 10;11(4):988. doi: 10.3390/microorganisms11040988 (PMC10146784; doi:10.3390/microorganisms11040988)
Supplement: Supplementary file 1 [file microorganisms-11-00988-s001.zip › Supplementary Figures_S1, S2.pdf]

Chemical characterization and metagenomic identification of endophytic microbiome from South African sunflower (*Helianthus annus*) seeds

Fatai Oladunni Balogun[1], Abidemi Ojo[2], Errol Cason[3], Saheed Sabiu[1]*

*** Correspondence:** Corresponding Author: email@uni.edu

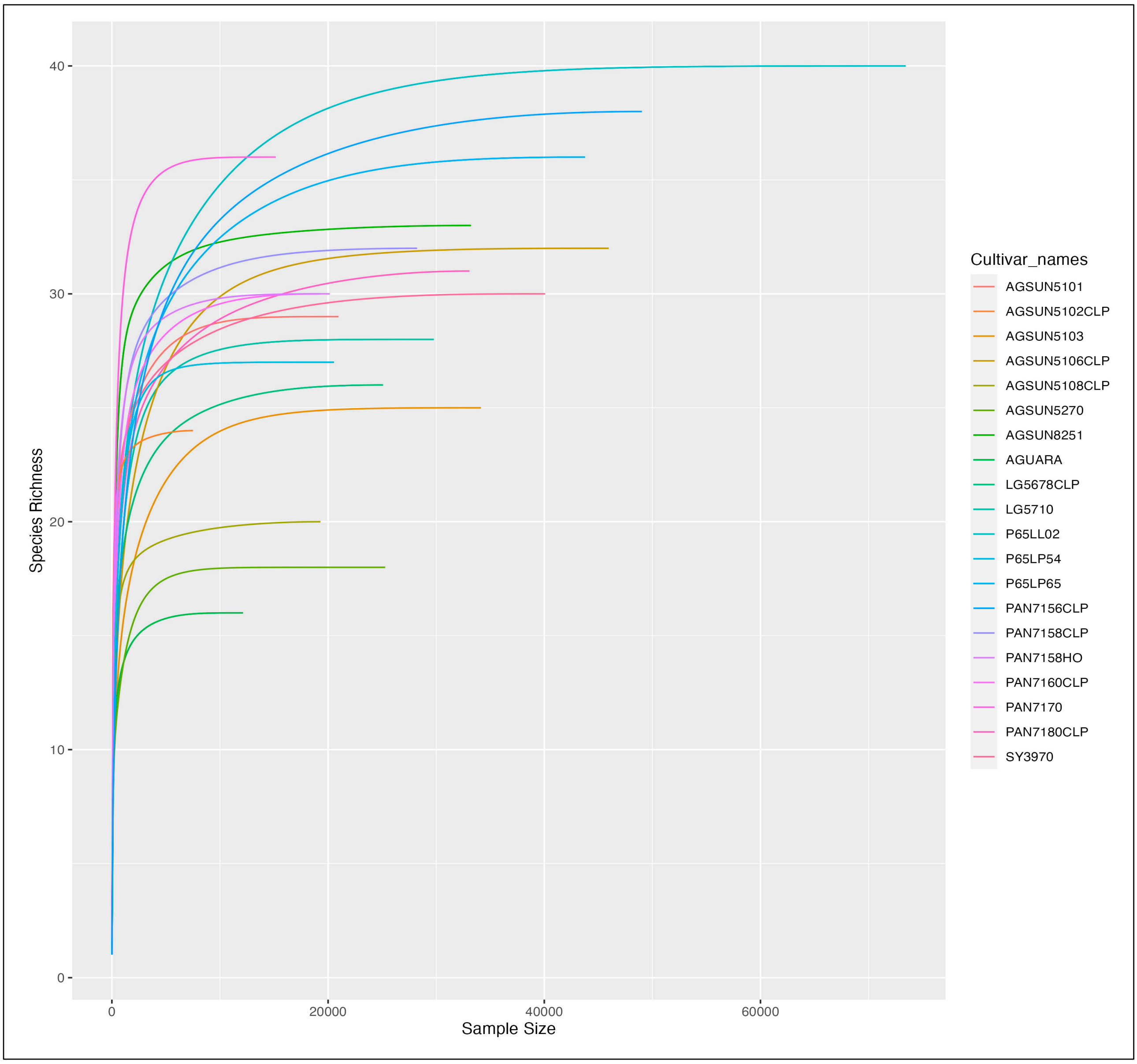

Figure S1: Rarefaction sequence curve of fungi diversity of sunflower seeds cultivars

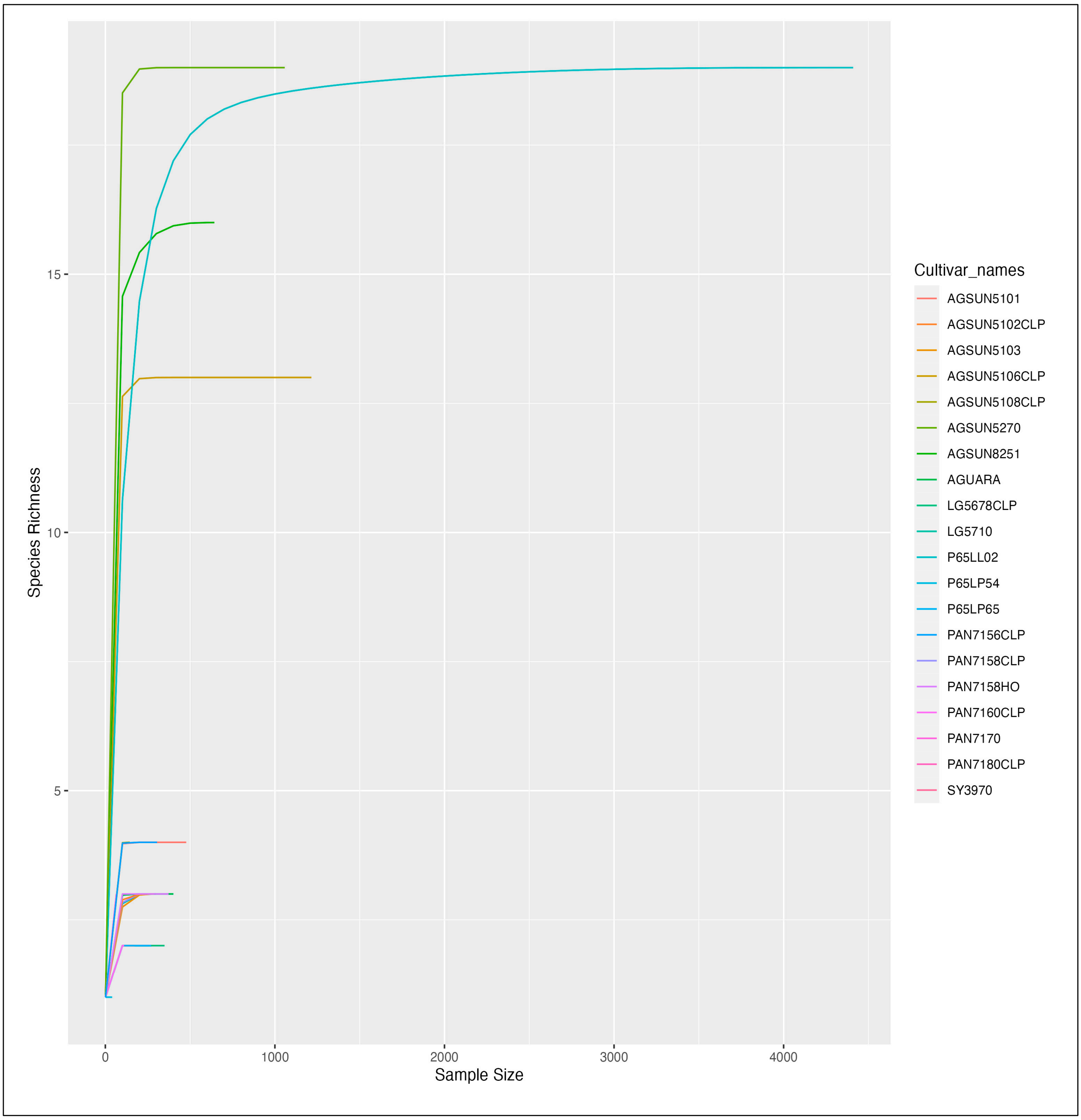

Figure S2: Rarefaction sequence curve of bacteria diversity of sunflower seeds cultivars
